# Assessing practical skills in cardiopulmonary resuscitation: Discrepancy between standard visual evaluation and a mechanical feedback device

**DOI:** 10.1097/MD.0000000000006515

**Published:** 2017-03-31

**Authors:** Baltasar Sánchez González, Laura Martínez, Manel Cerdà, Enrique Piacentini, Josep Trenado, Salvador Quintana

**Affiliations:** a Intensive Care Department, Hospital Universitari Mútua Terrassa. PhD program, University of Barcelona. Terrassa, Spain; b Consell Català de Ressuscitació. Barcelona, Spain; c Intensive Care Department, Hospital Universitari Mútua Terrassa. University of Barcelona. Terrassa, Spain.

**Keywords:** cardiopulmonary resuscitation quality, cardiopulmonary resuscitation simulation, cardiopulmonary resuscitation training, chest compressions quality, feedback from mechanical devices in CPR, inter-rater agreement

## Abstract

This paper aims to analyze agreement in the assessment of external chest compressions (ECC) by 3 human raters and dedicated feedback software.

While 54 volunteer health workers (medical transport technicians), trained and experienced in cardiopulmonary resuscitation (CPR), performed a complete sequence of basic CPR maneuvers on a manikin incorporating feedback software (Laerdal PC v 4.2.1 Skill Reporting Software) (L), 3 expert CPR instructors (A, B, and C) visually assessed ECC, evaluating hand placement, compression depth, chest decompression, and rate. We analyzed the concordance among the raters (A, B, and C) and between the raters and L with Cohen's kappa coefficient (K), intraclass correlation coefficients (ICC), Bland–Altman plots, and survival–agreement plots.

The agreement (expressed as Cohen's K and ICC) was ≥0.54 in only 3 instances and was ≤0.45 in more than half. Bland–Altman plots showed significant dispersion of the data. The survival–agreement plot showed a high degree of discordance between pairs of raters (A–L, B–L, and C–L) when the level of tolerance was set low.

In visual assessment of ECC, there is a significant lack of agreement among accredited raters and significant dispersion and inconsistency in data, bringing into question the reliability and validity of this method of measurement.

## Abstract 2

Supplemental Digital Content is available in the text

## 1 Introduction

Training generally involves assessing the knowledge and/or skills that students acquire. Assessment must be objective, a valid representation of the point being evaluated, and evaluator-independent. Although measurement always implies a certain degree of error, in an ideal testing situation, the only source of variation in a given measurement should be the variation among the individuals being tested. Nevertheless, various other sources of variation, such as intra- or inter-observer variation, are common, implying potential biases or increasing imprecision in the analysis of the phenomenon.^[1]^

The importance of training in cardiopulmonary resuscitation (CPR) is recognized not only for healthcare professionals, but also increasingly for other members of society.^[2]^ Ensuring CPR skills are correctly learned has more than mere academic significance; present evidence confirms strong associations between quality in CPR and cardiac arrest outcomes.^[3–5]^

Adequate training and evaluation are essential to ensure that CPR skills have been correctly acquired and can be translated into clinical practice. The first step in evaluating whether a set of skills has been acquired is to define the skills to be evaluated and the criteria for evaluating them. Given that high quality CPR is essential to improve outcomes, the 2010 European Resuscitation Council (ERC) Guidelines for Resuscitation established quality criteria for external chest compressions (ECC). These guidelines recommend that CPR providers ensure chest compressions of adequate depth (at least 5 cm but not >6 cm) with a rate of 100–120 compressions per minute, allowing the chest to recoil completely after each compression and minimizing interruptions during ECC.^[6,7]^

At present, practical skills in CPR are predominantly assessed visually by ≥1 observers. However, more objective methods of assessing these skills, such as CPR manikins with specific software,^[8,9]^ mechanical devices,^[10–12]^ etc, provide excellent accuracy and feedback and are becoming more widely used. Moreover, retrospective analysis of video recordings is increasingly being used in pediatric CPR training, and preliminary data supports the feasibility of this technique in assessing the quality of CPR in adults.^[13–15]^

The most common statistical techniques used to analyze agreement between different raters and between different methods of assessment are the intraclass correlation coefficient (ICC) for quantitative variables^[16]^ and Cohen's kappa (*K*) coefficient for qualitative variables.^[17]^ However, some authors consider Bland–Altman plots the best approach for evaluating agreement between 2 measurements,^[18]^ and others have recently proposed a new approach to assess the reliability of quantitative measurement, the survival–agreement plot.^[19]^

We hypothesized that there are differences in the accuracy in the evaluation of ECC skills among human raters using classical visual analysis and a mechanical feedback device with dedicated software.

We aimed to analyze the agreement in the assessment of ECC skills among 3 human raters using classical visual analysis and to compare their ratings against an assessment by dedicated software built into a CPR manikin.

## 2 Method

### 2.1 Study design

This descriptive observational study was done during 5 recertification courses in basic life support carried out in 2013. At the end of the course and as part of the assessment of CPR skills, 3 blinded raters (A, B, and C), qualitatively accredited by the European Resuscitation Council as instructors in basic and advanced CPR with extensive experience (>5 years’ teaching and evaluation of CPR courses), visually assessed each participant's ECC at the same time. The 3 instructors, selected from the Catalan Resuscitation Council instructor pool, participated voluntarily in the study. They rated all participants qualitatively (pass/fail) and quantitatively following the 2010 ERC guidelines’ recommendations for ECC in CPR.^[6,7]^

Potential participants were randomly selected from the group of transport technicians of a health transport company; 54 agreed to participate, collaborating voluntarily and freely in the study. Participants were informed that they would undergo a test on their skills in performing ECC according to the criteria of quality of the 2010 European Resuscitation Guidelines. The participants were certified in basic CPR training by the ERC. All participants had completed the last recertification course in the previous year, with an average interval of 5 months.

The study was carried out in different training areas in Barcelona within the framework of reaccreditation courses in basic CPR. The same material, manikins, evaluators, and evaluation criteria were always used.

The evaluation was carried out on a mannequin incorporating dedicated feedback software (ResusciAnne Advanced SkillTrainer, Laerdal Medical AS; Stavanger, Norway. Associated software: Laerdal PC Skill Reporting System v.2.3.0) (L).^[9,20]^ The manikin (L) was placed on a firm even floor to avoid inaccuracies in measuring compression depth and was connected to personal computer (see Images, Supplemental Content, which illustrate the information obtained from the manikin during the performance of ECC).

L awarded a score for each of the following 4 quality criteria for ECC on adults according to the 2010 guidelines^[6,7]^: the mean percentage of ECC with hands correctly positioned; the mean percentage of correct ECC within the recommended depth (compression depth >50 mm but <60 mm); the mean percentage of ECC with correct chest decompression (allowing the chest to recoil completely after each compression, minimizing interruptions, and taking approximately the same amount of time for compression as for relaxation); and the mean rate of compressions per minute (compared to the recommended 100–120 per minute).

Before the start of the study, L's accuracy in sensing compression was assessed using the LUCAS Chest Compression System (Physio-Control/Jolife AB, Lund, Sweden) with the 2010 ERC's algorithm for ECC in basic lifesaving.

Given L's accuracy and precision, we considered it the reference measure for the study. Raters were instructed to score using the same criteria as L, assigning a score (0–100) for each of the 4 ECC quality criteria. Raters were blinded to the scores awarded by L and by the other raters. Students were awarded a pass/fail grade for the quality of ECC on the basis of the mean of each rater's scores (0–100) for the 4 variables; scores >50 were considered a passing grade.

### 2.2 Statistical analysis

Quantitative variables are expressed as means (SD) and the qualitative variable (pass/fail) is expressed as a percentage.

To analyze the agreement among the 3 human raters’ scores and between each rater's score and L, we used the ICC for pairs of raters for quantitative variables and the *K* coefficient for the qualitative variable (pass/fail).

We used Bland–Altman plots to depict the degree of agreement, graphically representing differences between 2 measurements against their mean to show the mean difference and 95% limits of agreement.

Lastly, to assess the “clinical” importance of the differences between human raters and L, we constructed survival–agreement plots. This approach extends the analysis of agreement through a graph capable of expressing the degree of agreement or disagreement as a function of several limits of tolerance. On a survival–agreement plot graph, “failure” would lie exactly at absolute values of the observed differences between the observers. Thus, the *X*-axis represents the observed differences, and the *Y*-axis represents the proportion of cases with differences that are at least as large as the observed difference. There is a step function typical of a survival analysis, without censored data, with the *Y*-axis representing the proportion of discordant cases.^[19]^ This approach can complement the Bland–Altman method; it has the practical advantage of considering the magnitude of the differences observed, helping us to appreciate the practical importance of these differences.

We used SPSS (IBM Corp. Released 2010. IBM SPSS Statistics for Windows, Version 19.0. (IBM Corp., Armonk, NY) for all statistical analyses.

All students and raters gave their informed consent to participate in this study.

## 3 Results

A total of 54 participants (mean age, 37.2 [6.1] years; 41 [76%] men) were evaluated by A, B, C, and L. There were no missing data for any of the variables collected.

The raters’ mean scores were: A, 51.8 (26.8); B, 62.8 (27.6); C, 65.7 (20.3); and L, 55.1 (20.7).

Table 1 shows the results of the analysis of agreement between pairs of raters. The raw agreement ranged from 66% to 85%. The agreement (expressed as Cohen's K and ICC) was ≥0.54 in only 3 instances and was ≤0.45 in more than half.

**Table 1:**
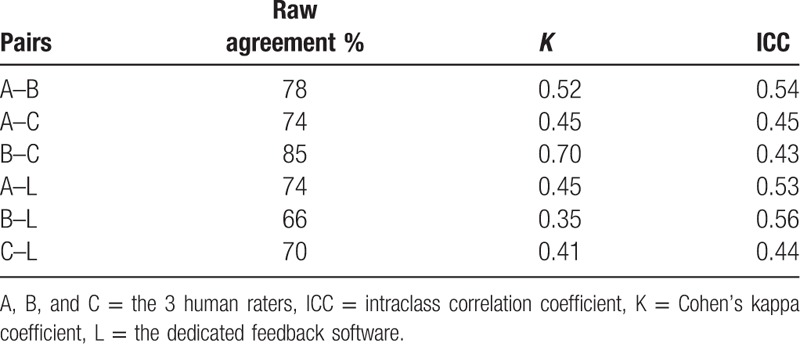
Raw agreement, Cohen's kappa coefficient, and ICC between pairs of observers.

**Figure 1:**
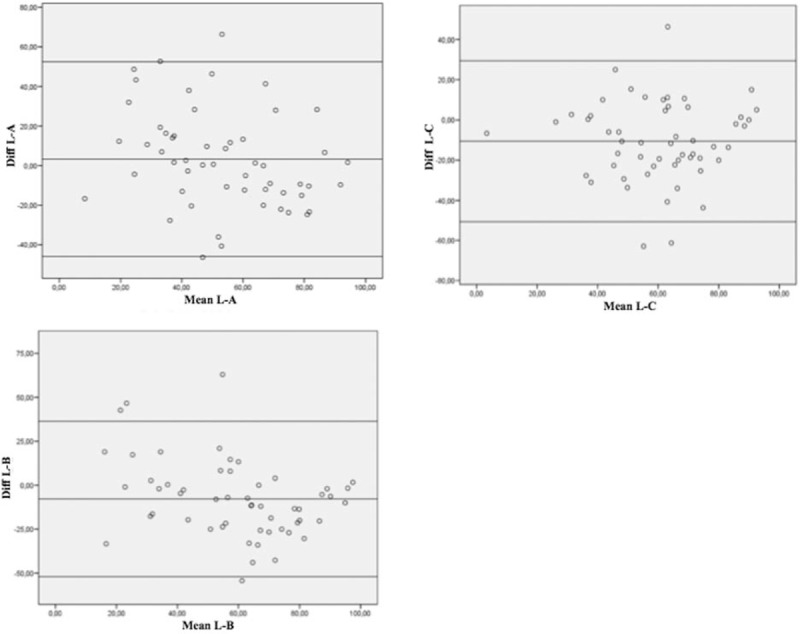
Bland–Altman plots. Legend: A, B, and C represent the 3 human raters, and L represents the dedicated feedback software. Diff L-A, Diff L-B, and Diff L-C represent the differences between the score (0–100) for each human rater versus L. Mean L-A, mean L-B, and mean L-C represent the mean score (0–100) between each human rater and L.

Figure 1 shows the Bland–Altman plot for each human rater versus L. On all plots, the values are dispersed, with means between –10 and 5, but 95% limits of agreement range from –55 to 50.

**Figure 2:**
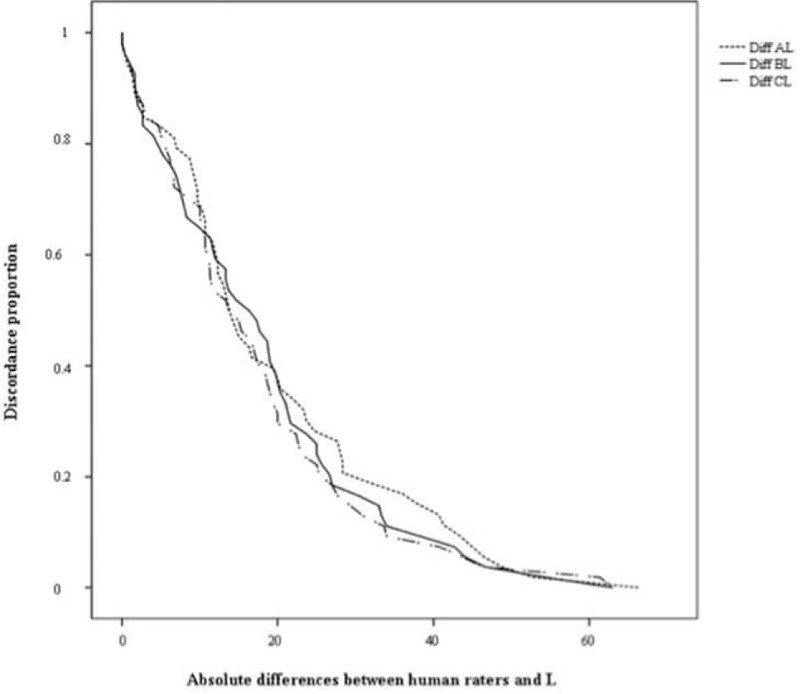
Survival–agreement plot: Proportion of discordance between students’ scores awarded by raters and L. Legend: A, B, and C represent the 3 human raters, and L represents the dedicated feedback software. Diff L-A, Diff L-B, and Diff L-C represent the differences between the score (0–100) for each human rater versus L. The *X*-axis (absolute differences between human raters and L) represents the observed differences, and the *Y*-axis (discordance proportion) represents the proportion of cases with differences that are at least as large as the observed difference and therefore represents the proportion of discordant cases.

Figure 2 shows the survival–agreement plot resulting from the agreement analysis of the scores (from 0 to 100) between pairs of raters (A–L, B–L, and C–L). The 3 curves are practically superimposed, showing a lack of concordance in each pair.

## 4 Discussion

We found a lack of agreement between human raters and the feedback device in assessing the quality of ECC.

It is difficult to ensure the reliability and validity of classical visual analysis of CPR skills because raters must measure various variables simultaneously. The aspects we chose for the instructors to assess were important for CPR quality and related to prognosis. Moreover, these aspects enabled us to provide clear and homogeneous rules for both students and raters, and the dedicated software provided a reliable, validated assessment reference system with which to compare instructors’ assessments.^[21]^Scant evidence is available about the accuracy of measurement with CPR devices; however, in a recent study, Beesems and Koster^[22]^ used a calibrated drill press to test the accuracy of measurement of compression depth on a similar Laerdal manikin model on different surfaces. Compared to the drill press, the manikin measured compression depth with an error <1 mm.^[22]^

We did not analyze all the information provided by L,^[9,23]^; we focused only on the evaluation of the quality criteria for ECC.^[6,7]^

Interobserver agreement refers to the consistency between ≥2 observers when evaluating the same measure. It is important to note that comparisons of average values (raw agreement) are not reliable and may lead to erroneous conclusions. In our study, the mean scores ranged from 51.8 to 65.7, and raw agreement was higher than 70% for most comparisons. However, the lack of large differences in mean scores assigned by different raters does not guarantee agreement, as if raters give high or low values oppositely, the mean value is not affected. When we adjusted for the probability of random agreement by using Cohen's kappa coefficient and the ICC, the agreement was poor. The ICC expresses agreement on a scale ranging from 0 to 1. The closer to 0, the greater the probability that the observed difference is owing to chance; conversely, the closer to 1, the greater the certainty that the observed variability is because of a true difference between subjects rather than to differences in measurement methods or observers. Coefficients ≥0.7 represent good agreement and coefficients <0.5 represent mediocre or poor agreement.^[24,25]^ The ICC was ≤0.45 in more than half the observations in our study.

Moreover, the Bland–Altman plots^[18]^ (Fig. 1) all show wide dispersion, consistent with the poor agreement found with Cohen's kappa coefficient and the ICC. No trend (e.g., an increase or decrease in the differences between values in relation to the mean) can be appreciated, indicating that these differences are not homogeneous.

Luiz et al^[19]^ proposed a new approach to assess the reliability of a quantitative measurement, survival–agreement plots. In Fig. 2, each of the 3 curves represents the comparison of a human rater's assessment against the L; the trend is similar for all 3 raters. The *X*-axis represents the observed differences between the human rater and L (e.g., 30 represents a difference of 30 points between the human rater's score and L's score in the evaluation of a student over a maximum of 100 points). The Y-axis represents the proportion of cases in which the difference was at least as large as the observed difference (i.e., the proportion of discordant cases, where 0 represents total agreement and 100 absolute disagreement).

Survival–agreement plots have the advantage of allowing us to visualize the data in function of the degree of difference in measurements we consider acceptable (i.e., in function of several levels of tolerance). For instance, 90% agreement between the human rater and L occurs only when differences in scores reach >40 points over 100. On the other hand, if the maximum difference we are willing to accept between the human rater's and L's score is 10 points over 100, the degree of disagreement between the rater and L would be ∼70%.

To our knowledge, this is the first study to evaluate the agreement between human raters and dedicated software in the assessment of the quality of ECC in the context of adult CPR training, although Hsieh et al^[26]^ recently analyzed the agreement between assessment by a mechanical device (QCPR Viewer^[23]^), Laerdal Medical,^[9]^ and human raters’ visual assessment of video-recorded ECC in pediatric CPR. Our results corroborate their findings of poor agreement about depth and release. However, we disagree with their conclusion that visual analysis is an accurate method for determining ECC compression rate. We consider that being able to see the respiratory rate monitor (a translation of chest pressure wave from the ECC) probably helped bring raters’ assessments closer to those of the mechanical device. On the other hand, their study used Pearson correlation rather than statistics normally used to assess agreement.^[16–18]^

Given the direct relationship between the quality of CPR and survival,^[4,27,28]^ CPR training requires appropriate assessment of the skills acquired. Our study shows that classical evaluation by human observation does not have adequate agreement with the more accurate assessment by a dedicated mechanical system.

We found large discrepancies both among the human raters and between each rater and L, casting doubt on human instructors’ ability to provide feedback and to assess CPR skills. Thus, it seems impossible to ensure that expert instructors’ visual assessments are a valid, accurate, reliable, representative, and evaluator-independent method to analyze students’ skills.

Mechanical devices such as the manikin used in our study with audiovisual feedback^[29]^ and others such as QCPR^[23]^ or CPR meter^[20]^ ensure accurate feedback about skills, enabling corrections and improvements that help guarantee correct training, a necessary first step in the acquisition of these skills and their translation to clinical practice. Devices that provide audiovisual feedback are also useful in human CPR. Other benefits of these devices are improved analysis of each of the assessed skills and the ability to make comparisons within and between students, making it easier to detect weak points and areas for improvement.^[21]^ To ensure the greatest translation of the skills acquired to clinical practice, we recommend using appropriate feedback devices for training and evaluation in CPR and striving to improve the homogeneity between different evaluators in the visual analysis through adequate training and clear and measurable objectives.

## 5 Limitations

The quality of our sample of students could be considered a limiting factor. Study participants were trained personnel; the same exercise performed by untrained individuals might result in different scores and concordance between raters.

Given the absence of bibliographical reference in relation to the subject of our study and its preclinical and merely descriptive character, a previous calculation of sample size was not performed.

Using manikins instead of real victims could be another limiting factor; however, all participants were regularly practicing CPR in real life, so we would expect little changes from real practice.

The translation of our results to clinical practice would require studies designed for this purpose.

## 6 Conclusion

Our study of the visual assessment of external chest compressions by accredited raters found a significant lack of agreement among raters and wide dispersion and inconsistency of data, calling into question the reliability and validity of this approach.

## Supplementary Material

Supplemental Digital Content
