# Supplementary material for: Assessing practical skills in cardiopulmonary resuscitation: Discrepancy between standard visual evaluation and a mechanical feedback device
Source: Medicine (Baltimore). 2017 Mar 31;96(13):e6515. doi: 10.1097/MD.0000000000006515 (PMC5380293; doi:10.1097/MD.0000000000006515)
Supplement: Supplemental Digital Content [file medi-96-e6515-s001.pdf]

Assessing practical skills in cardiopulmonary resuscitation: discrepancy between standard visual evaluation and a mechanical feedback device

First Author: Baltasar Sánchez [a,b]

[a]Intensive Care Department, Hospital Universitari Mútua Terrassa. PhD program, University of Barcelona. Terrassa, Spain.

[b]Consell Català de Ressuscitació. Barcelona, Spain.

Supplemental Digital Content

Manikin:

The evaluation was carried out on a manikin incorporating dedicated feedback software (Resusci®Anne Advanced SkillTrainer, Laerdal Medical AS; Stavanger, Norway. Associated software: Laerdal PC Skill Reporting System v.2.3.0) (L).

These are images obtained from the corporate website Laerdal ® (http://www.laerdal.com) about the manikin model and a similar type of software that shows how information is obtained from external chest compressions (ECC) performed on the manikin .

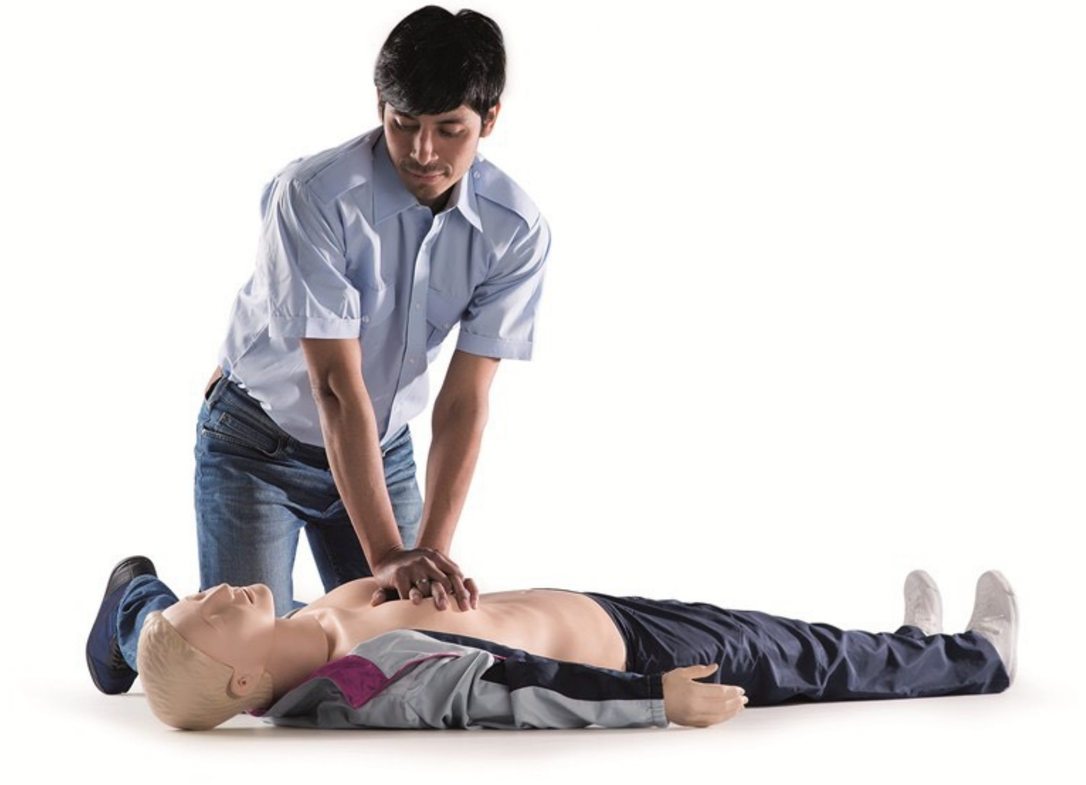

The following images show the information obtained from the manikin about the hands position, the depth, the rate and the chest decompression during the performance of ECC. Additionally information can be obtained about ventilation, a summary of the overall percentage of ECC performed properly and about each of the parameters listed above, the total time performed and the hands-free time.

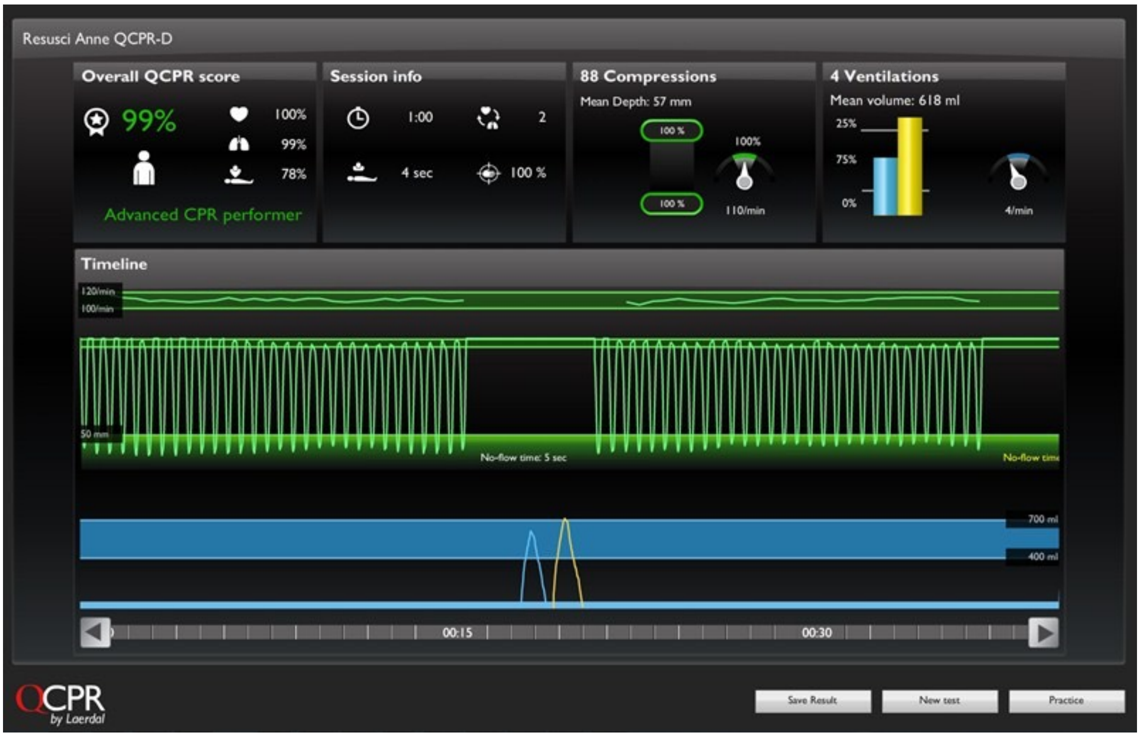
Resusci Anne QCPR-D
Overall QCPR score
99%
100%
99%
78%
Advanced CPR performer
Session info
1:00
2
4 sec
100 %
88 Compressions
Mean Depth: 57 mm
100 %
100%
100 %
110/min
4 Ventilations
Mean volume: 618 ml
25%
75%
0%
4/min
Timeline
120/min
100/min
50 mm
No-flow time: 5 sec
700 ml
400 ml
00:15
00:30
QCPR
by Laerdal
Save Result
New test
Practice

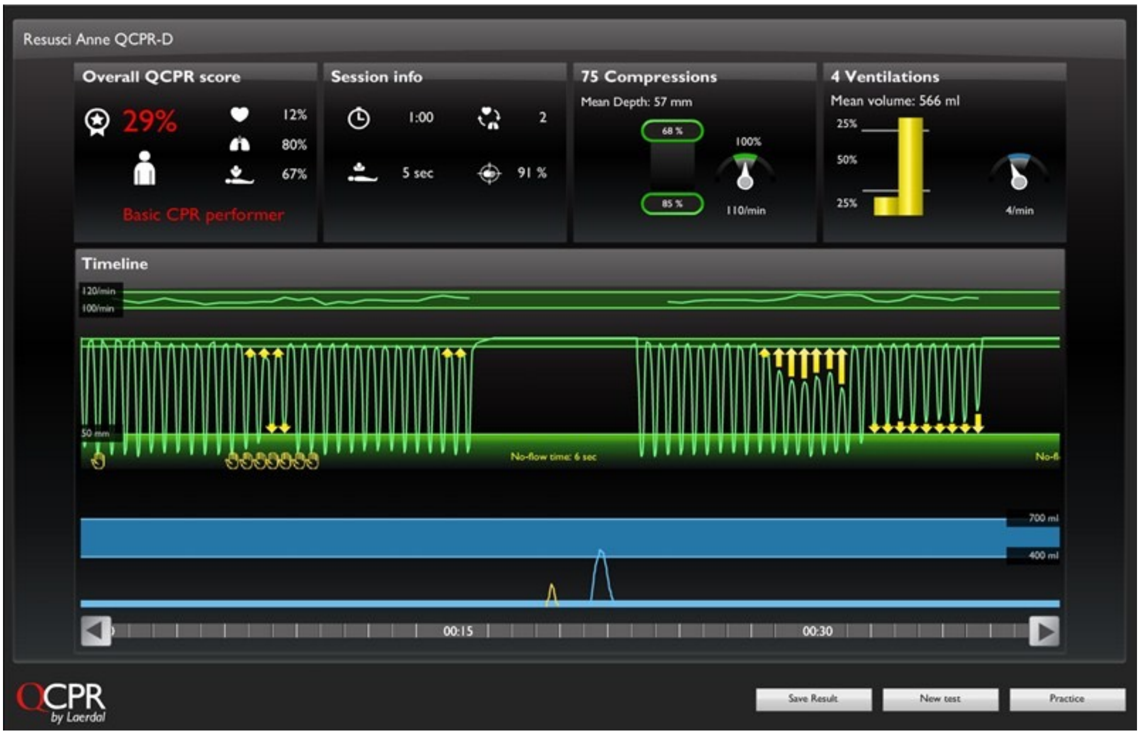
Resusci Anne QCPR-D
Overall QCPR score
29%
12%
80%
67%
Basic CPR performer
Session info
1:00
2
5 sec
91 %
75 Compressions
Mean Depth: 57 mm
68 %
100%
85 %
110/min
4 Ventilations
Mean volume: 566 ml
25%
50%
25%
4/min
Timeline
120/min
100/min
50 mm
No-flow time: 6 sec
700 ml
400 ml
00:15
00:30
QCPR
by Laerdal
Save Result
New test
Practice
